# Female-limited X chromosome evolution reveals that lifespan is mainly modulated by interlocus rather than intralocus sexual conflict

**DOI:** 10.1007/s00265-022-03231-4

**Published:** 2022-08-17

**Authors:** Katrine K. Lund-Hansen, Megan A. M. Kutzer, Sophie A. O. Armitage, Samuel Gornard, Hamilcar Keilani, Jessica K. Abbott

**Affiliations:** 1 Biology Department, Lund University, Lund, Sweden; 2 Institute of Evolutionary Biology, University of Edinburgh, Edinburgh, Scotland; 3 Institute of Biology, Freie Universität Berlin, Berlin, Germany

**Keywords:** Resistance, Tolerance, *Drosophila melanogaster*, Sexual antagonism, Experimental evolution, Longevity

## Abstract

**Abstract:**

Sexual dimorphism in somatic investment may be shaped by two distinct forms of sexual conflict; under intralocus sexual conflict (IASC), males and females have different optimal levels of somatic investment but are constrained from reaching their respective optima by their shared genome, while under interlocus sexual conflict (IRSC), males and females have different optimal sexual strategies, which could have direct or indirect effects on levels of somatic investment. We investigated effects of IASC and IRSC on two aspects of somatic investment, immune defence strategies and longevity, using previously established female-limited experimental evolution lines in *Drosophila melanogaster*. We found little evidence for any effect of either type of sexual conflict on investment in the immune defence resistance or tolerance. Nor did we find convincing evidence that longevity is subject to IASC in this species. However, we did find evidence that increased female control over mating rate had important and opposite effects on longevity between the sexes. Specifically, females that had adapted to high levels of female control over mating had a longer lifespan when kept in mixed-sex groups, while males had shorter longevity, perhaps due to increased investment in post-copulatory sexual selection. These novel results show that female control over mating rates may have important and unexpected effects on patterns of somatic investment.

**Significance statement:**

Sexual conflict occurs between the two sexes over numerous life history traits, and it is complex to disentangle how these traits interact and affect each other. Here we use a long-term evolution experiment to investigate sexual dimorphism in somatic maintenance. We found no effect of feminising the X chromosome on female immune defence. However, we did find that increased female control over mating rate resulted in longer female lifespan, but reduced male lifespan, and that these effects were dependent on social context (isolated or in mixed-sex groups). Unlike previous studies on the effect of sexual conflict on longevity, our experiment did not manipulate environmental conditions nor the adult sex ratio, which is likely to reduce both pre- and post-copulatory sexual selection.

**Supplementary Information:**

The online version contains supplementary material available at 10.1007/s00265-022-03231-4.

## Introduction

In species with separate sexes, males and females are often exposed to different selection pressures and therefore have different phenotypic optima which selects for sex-specific adaptations (Parker 1979). A common pattern among sexually reproducing species is an increased investment in somatic maintenance in females compared to males. This investment may take various forms, including larger body size (Teder 2014), longer lifespan (Adler and Bonduriansky 2014), or increased investment in immune function (McKean and Nunney 2005, but see Nystrand and Dowling 2020). Consistent with this pattern, theory suggests that the sex that competes more intensely for access to mates (typically males) tends to be selected to pursue a reproductive strategy that promotes early and multiple reproductive opportunities (Adler and Bonduriansky 2014), but causes a higher mortality rate and accelerated senescence compared to the opposite sex (typically females; Vinogradov 1998). This results from the fact that potential short-term fitness payoffs available to males tend to be relatively large, as males have a higher maximum potential reproductive rate than females in most species (Adler and Bonduriansky 2014). These differences are expected to be particularly pronounced in promiscuous species where variance in fitness is larger in males than in females (Arnqvist and Rowe 2005; Fritzsche and Arnqvist 2013). Conversely, female reproduction is usually limited by resource availability (e.g. Kreiter and Wise 2001; Nagy and Holmes 2005) which means that investment in somatic maintenance may be more favourable if it increases resource acquisition.

Two different forms of sexual conflict are expected to shape sex-specific investment in somatic maintenance. Firstly, a single locus can be subject to conflicting selection if a shared trait has opposite fitness effects between the sexes. For example, in a species where female optimum lifespan is longer than male optimum lifespan, an allele which increases lifespan in both sexes will be positively selected in females, but negatively selected in males, resulting in so-called intra-locus sexual conflict (IASC; Bonduriansky and Chenoweth 2009; Schenkel et al. 2018). Under IASC, one or both sexes may be displaced from their phenotypic optimum by counter-selection in the opposite sex. In *Drosophila melanogaster*, there is evidence of ongoing IASC over several traits, such as adult locomotor activity (Long and Rice 2007), body size (Abbott et al. 2010), and growth rate (Prasad et al. 2007). Secondly, if different (and genetically independent) traits are favoured in each sex, then this can lead to so-called inter-locus sexual conflict (IRSC; Bonduriansky and Chenoweth 2009; Schenkel et al. 2018). For example, in *D. melanogaster*, the ejaculate contains accessory gland proteins which increase female fecundity and thereby increase the male’s reproductive fitness (Nandy et al. 2013). However, these proteins are also toxic to females and reduce their lifespan, so this reduction should lead to selection for resistance to remating in females in order to reduce exposure to the toxic accessory gland proteins. IRSC could therefore influence investment in somatic maintenance either by a trade-off between sexually selected and somatic traits, or by modulating the relative advantage associated with a given level of somatic investment.

Here we used experimental evolution to investigate how patterns of investment in somatic maintenance (immune defence and lifespan) change as a result of altered sex-specific selection pressures in *D. melanogaster*. Immune responses can be sexually dimorphic in this species (McKean and Nunney 2005; Siva-Jothy and Vale 2021; Vincent and Dionne 2021), and there is some evidence that immune defence can be subject to IASC (Vincent and Sharp 2014). In addition, upregulation of immune genes after mating Fricke et al. (2020) suggests that IRSC could be a contributing factor to the evolution of this sexual dimorphism (Fedorka et al. 2007; Innocenti and Morrow 2009, but see Short and Lazzaro 2010). Similarly, longevity is also sexually dimorphic and has been found to be subject to ongoing IRSC in *Drosophila* (Wigby and Chapman 2004; Nandy et al. 2013; Arbuthnott et al. 2014; Duxbury et al. 2017), and results from other species suggest that it could potentially be subject to IASC as well (e.g. Berg and Maklakov 2012). We therefore used a female-limited X chromosome evolution experiment to determine if release from IASC and/or IRSC could affect one or both traits. The genetic contribution of the X chromosome to traits associated with sexual conflict is particularly interesting because it has sometimes been predicted to be a hotspot for IASC (Rice 1984; Gibson et al. 2002) and may play a disproportionately large role in the evolution of sexual dimorphism since it spends 2/3 of its time in females and 1/3 in males (Rice 1984; Dean and Mank 2014).

In our experimental setup, the X chromosome experienced female-limited selection, which should release females from counter-selection in males, and select for phenotypic feminisation in both sexes (Lund-Hansen et al. 2020). We therefore expected to see an altered immune defence and a shift towards longer lifespan in the female-limited X chromosome selection regime, as a result of release from IASC. However, the experimental protocol that we used to enforce matrilineal inheritance of the X seems to have altered sexual conflict dynamics by increasing female control over mating rate (Lund-Hansen unpublished data, Manat 2021), which allowed us to also investigate how somatic maintenance is modulated by the intensity and/or form of IRSC. Predictions about the response to altered IRSC are less straightforward, but since male adaptations to sexual conflict seem to increase female mortality (Rice 1996; Arnqvist and Rowe 2005) and lead to higher rates of senescence (Promislow 2003; Maklakov et al. 2007), we expected that altered IRSC in the methodological control selection regime (see methods) would result in increased lifespan in one or both sexes. The relationship between post-mating immune response and survival when infected by pathogens is unclear (Oku et al. 2019), so we did not have any definite hypothesis how increased female control over mating rates in the methodological control regime would affect the immune defence. However, we expected that a response could be possible through changes in resistance (the ability to limit pathogen burden), tolerance (the ability to resist harm induced by pathogen infection), or both aspects of immune defence (Råberg et al. 2007; Kutzer and Armitage 2016a). Indeed, although there was no evidence of any change in immune defence, we did find evidence of changes in longevity consistent with altered IRSC dynamics. Because the experimental protocol does not result in any direct selection on survival beyond 14 days, these changes are likely the result of altered patterns of investment in somatic maintenance relative to pre- and post-copulatory sexual selection.

## Methods

### Selection regimes

The experimental evolution populations used in this paper were established in 2013 from an LH_M_ base population and maintained under the standard LH_M_ protocol (25 °C, 12/12 light/dark cycle, 60% relative humidity) and fed on cornmeal-molasses-yeast medium (Rice et al. 2005).

The evolution experiment consists of three regimes, one selection regime and two control regimes, in four replicated populations each. In the female-limited X chromosome (FLX) selection regime, X chromosomes are forced to only be inherited from mother to daughter, thereby removing any male-specific selection on the X. Matrilineal inheritance of the X was ensured via the use of an X chromosome balancer (FM7a), which does not recombine with the normal X. The FM balancer carries a number of phenotypic markers which make it possible to identify individuals who are homozygous or heterozygous for the balancer, including the bar eye phenotype which renders males (and female homozygotes) blind. A methodological control regime, control FM (CFM), was therefore included to control for any unforeseen effects of the balancer chromosome. In this treatment, females are heterozygous for the balancer (the same as in the FLX treatment), but X chromosomes spend every third generation in males (resulting in the normal pattern of 2/3 of the time in females and 1/3 of the time in males). Finally, we also included a standard wildtype control regime (Cwt). A detailed description of the experimental evolution set-up can be found in Lund‐Hansen et al. (2020), and see SI Fig. 1 for an overview. Differences between the Cwt regime and the two other selection regimes are therefore expected to be a result of effects of adaptation to the presence of the balancer chromosome, while differences between the FLX and CFM regimes are expected to be a result of release from IASC in the FLX selection regime.

**Fig. 1:**
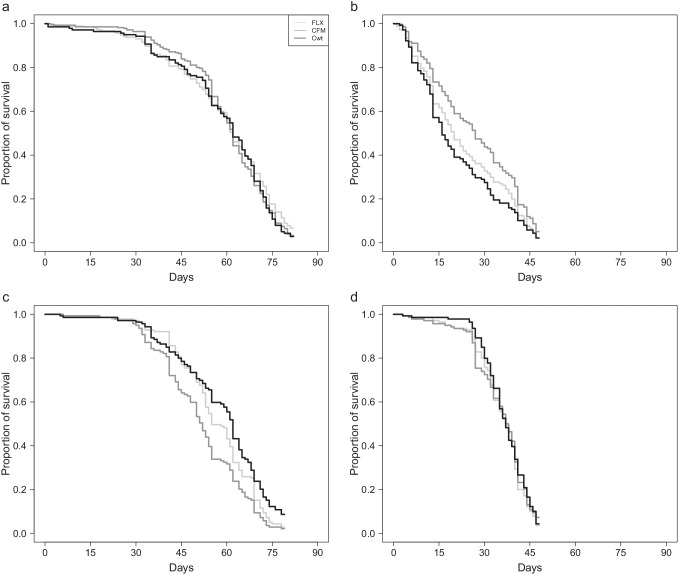
Lifespan for the different selection regimes in both sexes either isolated or in mixed-sex groups. Light grey line: FLX regime, dark grey line: CFM regime, and black line Cwt regime. **a** Female lifespan when isolated. We did not find any significant difference in lifespan between the three regimes when kept isolated. **b** Female lifespan in mixed-sex groups. We did find a significant difference in lifespan between the three regimes when the females were continuously exposed to males (*p* = 0.030). **c** Male lifespan when isolated. We found a significant difference in lifespan between the three regimes when the males were kept isolated (*p* = 6.765e^−05^). **d** Male lifespan in mixed-sex groups. There were no significant different in lifespan between the three regimes, when the males could continuously mate with females

### Immune defence assay

This assay was carried out at generation 48. All target flies for the immune function assay were kept at 25 °C, 70% relative humidity on a 12–12-h light–dark cycle. Virgins of both sexes from each regime were collected over three days and placed on a sugar-yeast-agar (SYA) medium (1.5% agar, 5% sugar, 10% yeast, 3% nipagin, and 0.3% propionic acid) in single-sexed groups of 20. At 3 to 4 days post adult eclosion, we placed the flies together in groups of 20 pairs for approximately 1 h to allow them to mate and then separated them using light CO_2_ anaesthesia. The matings were set up in a randomised order with respect to the identity of the selection populations. This procedure was carried out on successive days, resulting in two experimental replicates, each containing all twelve populations. Forty females per regime per replicate population and experimental replicate were placed in vials in groups of 10 in preparation for infections the following day.

### Bacterial preparation and infection

We infected the target females with the Gram-positive bacteria species, *Lactococcus lactis* (gift from Brian Lazzaro) or the Gram-negative species, *Pseudomonas entomophila* (gift from Bruno Lemaitre)*.* Each strain was isolated from wild-caught flies and were chosen because they differ from each other in gram-type and virulence, and because wildtype hosts have shown genetic variation in resistance and tolerance when infected with either species (Kutzer et al. 2018). Bacteria aliquots were stored at − 80 °C in glycerol. Two days before infections, *L. lactis* was plated out on lysogeny broth (LB) agar and *P. entomophila* was plated out on LB agar containing 1% milk so we could select for protease-positive clones (Neyen et al. 2012). We carried out bacterial preparation and infections following the protocol in Kutzer and Armitage (2016b) using a randomised block design (4 experimental treatments—Naïve, Ringer’s injected, *L. lactis*, *P. entomophila*—per regime per replicate population) with a total of 960 individuals, i.e. 480 per experimental replicate, kept in groups of five. We injected 18.4 nL of each bacteria solution or control solution into the left lateral side of the thorax using a Nanoject II (Drummond). *L. lactis* infected flies were injected with a 1 × 10^8^ cells mL^−1^ bacterial solution, which was equivalent to ~ 1840 bacteria per fly. *P. entomophila* infected flies were injected with a 5 × 10^6^ cells mL^−1^, which was equivalent to ~ 92 bacteria per fly. Ringer’s injected flies were injected with an equivalent volume of control solution and naïve flies were anesthetised and left untouched. All experimental flies were returned to 25 °C, 70% relative humidity following the injections. We diluted the remaining bacteria solution aliquots to confirm the injection dose and plated out the Ringer’s solution to look for evidence of contamination. We found no evidence of contamination in either experimental replicate.

### Survival and resistance assays

We followed survival for 7 days post infection in all experimental treatment groups, checking survival every 24 h. After 7 days, we homogenised all living flies in the *L. lactis* and Ringer’s injected treatment groups and then plated the homogenate on LB agar to quantify bacteria load, as a measure of resistance, and to confirm that our injection controls were clear of any secondary infection. We assayed bacteria load following the methods described in Kutzer and Armitage (2016b). However, we pooled the survivors from each vial instead of homogenizing individual flies. We incubated the LB plates at 30 °C for 20 h and then counted colony-forming units (CFUs) to determine bacteria load. The CFUs were then divided by the number of flies that had been homogenised, to calculate the mean number of CFUs per fly. Survival was monitored in the two remaining treatment groups, naïve and *P. entomophila* infected, until 22 days post-infection.

### Longevity assays

These assays were started at generation 189. All longevity assays were started at the same time, with both sexes being assayed separately in isolated (assay 1) and in mixed-sex groups (assay 2). All flies were collected as virgins before the start of the experiment, and to synchronise the assays, they were all started on day 14 after oviposition (i.e. approximately 4 days after eclosion). Thirty-five flies per regime, replicate population, and sex were included in each of the longevity assays.

### Longevity assay 1, isolated

The 35 virgin flies were collected and kept individually in test tubes throughout the experiment with the cornmeal-molasses-yeast food medium. The flies were flipped into fresh test tubes approximately every 10^th^ day. Mortality of the flies was scored daily, 6 days a week, until 95% of the target flies were dead.

### Longevity assay 2, mixed-sex groups

Five virgin flies in seven vials with standard medium were combined with five LH_M_-*bw* flies of the opposite sex on day 14 after eclosion. The LH_M_-*bw* stock is an outbred LH_M_ population homozygous for the visible brown eye (*bw*) genetic marker. The flies were flipped into fresh vials every third day, and new LH_M_-*bw* flies of both sexes were added to the vials to replace the dead flies and keep the sex ratio consistent throughout the assay. The LH_M_-*bw* flies were between 14 and 19 days old after oviposition. Mortality of the flies was scored daily, 6 days a week, until 95% of target flies were dead.

### Statistical analysis

All statistical analyses were conducted in R version 4.0.5 (R Core Team 2021). Resistance was analysed by fitting a linear mixed model (Bates et al. 2015) with bacterial load as the dependent variable, and regime and experimental replicate as fixed factors. We also included replicate population as a random effect nested within regime to avoid pseudoreplication. Survival tolerance was analysed by fitting a linear mixed model with survival proportion as the dependent variable, and regime, bacterial load, and their interaction, as well as experimental replicate as fixed factors; an interaction between bacterial load and regime would indicate variation in survival tolerance. We also included replicate population as a random effect nested within regime. Survival of the infected flies after 7 and 22 days was analysed using a cox proportional hazards model (coxme) fitted with an interaction between regime and experimental treatment (Naïve, Ringer’s injected, *L. lactis*, *P. entomophila*) as fixed factors as well as experimental replicate (Therneau 2020). Replicate population nested within selection regime and vial were also included as random factors.

Although all longevity assays were carried out at the same time, there is a large difference in survival rates between continually mated and virgin males and females (Fowler and Partridge 1989; Cordts and Partridge 1996), we therefore analysed the data separately by assay type and sex. Longevity was analysed using a cox proportional hazards model (coxme) fitted with regime as a fixed factor and replicate population nested within selection regime as a random factor. In the mixed-sex group analysis, vial was also included as random factor. Tukey post hoc tests were done for any effects that were significant (Hothorn et al. 2010).

## Results

### Female immune defence

There was no evidence of a difference in resistance to infection with *L. lactis* between the three selection regimes (*F*_2_ = 0.38, *p* = 0.697, SI Table 1, SI Fig. 2a). Neither was there a significant difference in survival tolerance to *L. lactis* infection between the three regimes (*F*_2_ = 0.56, *p* = 0.574 SI Table 1, SI Fig. 2b). We then followed the survival of the four different experimental treatments and found no significant difference after 7 days (χ^2^(6) = 3.75, *p* = 0.711, SI Table 2, SI Fig. 3a), and no significant difference between naïve and *P. entomophila* injected flies after 22 days (χ^2^(2) = 3.83, *p* = 0.148, SI Table 2, SI Fig. 3b). However, plots of the survival differences between selection regimes within each experimental treatment suggested a possible difference in the naïve flies (SI Fig. 3b). We therefore analysed the naïve flies separately and found a trend towards a significant difference in survival rates between the three selection regimes (χ^2^(2) = 4.72, *p* = 0.095, SI Table 2 & 3, SI Fig. 3c), with CFM females having the highest survival rate. The longevity assays were later designed to follow-up on this result. See the supplementary information for full statistical details (SI Tables 1–3).

**Table 1:** Summary of the significant ANOVA results for the Cox mixed-effects models for the flies either isolated or in mixed-sex groups

Source	*df*	*χ*^*2*^	*P*
Longevity female mixed-sex group			
Regime	2	7.04	0.030
Longevity male isolated			
Regime	2	9.10	0.011

### Female longevity

We measured female longevity under two different conditions, isolated and mixed-sex groups. When the females were kept isolated, we found no significant difference in longevity between the three selection regimes (χ^2^(2) = 0.46, *p* = 0.796, Fig. 1a, SI Table 4 & 5). We did, however, find a significant difference in longevity between the three regimes when the females were kept in mixed-sex groups (*p* = 0.030, Fig. 1b, Table 1, and SI Table 6), with CFM females having a significantly higher survival rate than the Cwt females (CFM-Cwt: *p* = 0.026), and FLX being intermediate (SI Table 7).

### Male longevity

Male longevity was also measured, isolated, and in mixed-sex groups, and interestingly, we found the opposite pattern to that seen in female longevity. There was a significant difference in longevity between the three selection regimes when the males were assayed alone, (*p* = 0.011, Fig. 1c, Table 1, and SI Table 8), but not when they were assayed in mixed-sex groups (χ^2^(2) = 0.76, *p* = 0.685, Fig. 1d, SI Table 3 & 9). In contrast to females where the CFM selection regime had the highest longevity, in males, we found that the CFM selection regime had a significantly lower survival rate compared to Cwt, while FLX was again intermediate (CFM-Cwt: *p* = 0.007, SI Table 7).

## Discussion

Our results were partially consistent with our predictions. Despite our expectations, there were no differences between the selection regimes in any measure of immune defence. However, we did find differences between selection regimes in longevity in both sexes, and that the differences were dependent on social context (i.e. isolated or in mixed-sex groups). We discuss these results below in light of theories about IASC and IRSC.

### Female immune defence

For immune defence strategies, we expected to see a change in tolerance and/or resistance in FLX females compared to CFM females if immune defence is mainly shaped by IASC (Vincent and Sharp 2014), or between Cwt and CFM females if immune defence is mainly shaped by IRSC (Short and Lazzaro 2013). There could be several reasons why we did not detect any differences between the selection regimes. Firstly, the immune defence is often context-dependent and may be influenced by both social factors and choice of pathogen (Kutzer and Armitage 2016b; Leech et al. 2019). It is therefore possible that differences exist, but we were unable to detect them due to our choice of experimental design. Secondly, the evidence for X-linked immune genes is mixed and again depends on the context and the immune trait investigated (Hill-Burns and Clark 2009; Arun et al. 2020). There may therefore be few X-linked loci which affect immune defence, in which case the FLX selection regime would not be effective in producing a response arising from release from IASC. Finally, it is possible that immune defence strategies are not affected by either IASC or IRSC in the ancestral population. These explanations are of course not mutually exclusive, and we cannot know from the data at hand which explanation is the correct one.

### Female longevity

If longevity is subject to IASC in this species in the way that it has been demonstrated to be in some others (e.g. Berg and Maklakov 2012), then we would expect to find increased longevity in the FLX selection regime as a result of release from IASC. However, contrary to this prediction, the largest difference in female longevity was between the CFM and Cwt regimes, rather than between the FLX regime and the others (Fig. 1b). This suggests that the differences in longevity we observed cannot simply be explained by the feminizing effects of the FLX selection regime, and that a more plausible explanation is altered IRSC. Previous results suggest that the FLX and CFM regimes have been subject to a change in sexual conflict dynamics as a side-effect of using the FM balancer (Lund‐Hansen et al. 2020; Manat 2021). FM males have lower reproductive fitness than wild-type males (Lund‐Hansen et al. 2020), which appears, largely, to be driven by their lack of chase behaviour during courtship (Lund-Hansen, unpublished data). We believe that this lack of an important courtship behaviour may have shifted the control over mating rates to females, as the females now have to be the initiator of mating by approaching a male and staying close to him throughout courtship. Such a shift in control over mating rate should reduce the overall level of IRSC experienced by females in the CFM and FLX selection regimes, and likely shift male-male competition from the pre- to the post-copulatory arena in these regimes. This interpretation is supported by transcriptomic data, which found signatures of changes in expression of genes previously found to be associated with sexual conflict (Manat 2021). Additional evidence that there have been alterations in IRSC dynamics in the CFM and FLX selection regimes comes from the fact that the differences in longevity among selection regimes in females were only apparent when the flies were kept in mixed-sex groups (compare Fig. 1a and b). This suggests that adaptation to altered sexual conflict dynamics has changed female investment in somatic maintenance, but that the effect of this altered investment is mediated by interactions with males. We therefore hypothesise that increased female control over mating enabled the CFM females to increase their allocation to somatic maintenance, resulting in increased lifespan in this selection regime compared to the Cwt females, when continuously exposed to males.

Assuming that this hypothesis is true, why then was the FLX regime intermediate between the CFM and Cwt selection regimes? Since both the FLX and CFM selection regimes had the opportunity to adapt to the altered IRSC associated with the presence of the FM balancer, then if this was the only force at play, we would expect to see similar increases in longevity in both selection regimes. Although it is possible that the release from IASC we expected in the FLX regime could be a factor, we do not feel this is likely. Release from IASC should also result in increased longevity, reinforcing the effect of release from IRSC rather than mitigating it; so under a combined IASC + IRSC scenario, the FLX regime should therefore have had the highest longevity, with CFM being intermediate. Instead, our hypothesis is that exposure to normal males is the key. In the CFM regime, females are exposed to wild-type males once every third generation, while the FLX females are never exposed to wildtype males. Relaxed selection on female resistance to males in the FLX regime (e.g. to male mating harassment or toxic ejaculate compounds) may have therefore reduced their longevity when exposed to wild-type males, relative to the CFM females, in line with previous results (Wigby and Chapman 2004; Nandy et al. 2013; Arbuthnott et al. 2014; Duxbury et al. 2017).

Conversely, if only a relaxation of female resistance were in play, we would expect Cwt females to be to the most resistant and therefore least impacted by continuous exposure to males, resulting in a pattern in which the FLX females would have the lowest longevity, CFM would have intermediate longevity, and Cwt would have the highest longevity. As we did not observe this pattern, other factors must be in play. Apart from differences in resistance, it is possible that the non-significant difference in longevity between FLX and Cwt females is due to other changes as a result of the FLX evolution experiment, since we have previously shown that other traits which may have an impact on longevity have shifted towards the female optimum in the FLX selection regime. Specifically, FLX females have been shown to be significantly larger than Cwt females (Lund‐Hansen et al. 2020), and body size has been shown to be positively correlated with longevity (Norry and Loeschcke 2002). Also, being larger than the males can help facilitate female rejection behaviour during courtship (Pitnick and García–González 2002), and thus lessen the negative impact of male harassment for the FLX females. This difference may also have been exacerbated by the fact that all the selection regimes have likely experienced selection for decreased development time, since all experimental females are collected as virgins. This may have resulted in negative correlated effects on longevity in the Cwt females, which were countered to a greater or lesser extent in the FLX and CFM females.

### Male longevity

Interestingly, the results from males were opposite to those from females in two ways; firstly, differences between the regimes were only apparent when males were kept in isolation, and secondly, CFM males had the shortest longevity, and Cwt males had the longest longevity (Fig. 1c). Again, this suggests that this response is mainly due to the effects of altered IRSC rather than release from IASC, and that increased female control over mating has resulted in decreased male investment in somatic maintenance in the CFM regime. Although we cannot be sure from the data at hand why this was the case, one plausible mechanism is increased investment in post-copulatory mating success at a cost to longevity. When given the opportunity, females often mate multiply in this species, so increased female control over mating is likely to have shifted male-male competition towards the post-copulatory area. Indeed, sperm competition data from the selection regimes suggests increased investment in sperm offense (i.e. displacement of other males’ sperm) in CFM males compared to Cwt and FLX males (Manat et al. 2021). Assuming that males adjust their investment in pre-copulatory sexual selection according to their own condition and the risk of sperm competition (Bretman et al. 2010; Narayan and Wang 2021), this could explain why there were no differences between the regimes in longevity when males were kept in mixed-sex groups—any energy the Cwt and FLX males “saved” compared to the CFM males when kept in isolation could have been reallocated to pre-copulatory success when kept in mixed-sex groups.

Here again, we might wonder why the FLX selection regime was intermediate between the other two. Under an IASC + IRSC scenario, we might expect to see the observed pattern of differences, i.e. that if increased investment in postcopulatory sexual selection results in lower longevity in CFM males, this could be partially countered by phenotypic feminisation (increased longevity) in the FLX selection regime, leading to intermediate longevity in FLX males compared to CFM and Cwt males. However, we do not think that this is a likely explanation. Release from IASC should result in the fixation (or at least increased frequency) of alleles which are beneficial to females and detrimental to males (Bonduriansky and Chenoweth 2009). In the context of longevity, this would be alleles which increase longevity in both sexes, assuming the female optimum longevity is longer than the current female mean, and the male optimum longevity is shorter than the current male mean. Any phenotypic effects of release from IASC should therefore be similar in both sexes. Since the FLX selection regime had a shorter longevity than the CFM selection regime in females (Fig. 1b), but a higher longevity than the CFM regime in males (Fig. 1c), this suggests that IASC has not played a major role in influencing changes in longevity among the selection regimes.

Although the results for the mixed-sex groups may seem counterintuitive to the general rule that females live longer than males, mating is known to decrease the lifespan of both males (Partridge and Farquhar 1983) and females (Partridge et al. 1986) in *D. melanogaster*. To our knowledge, no studies to date have specifically investigated which sex experiences a greater reduction in longevity per mating, but this study is not the first to report shorter longevity in females compared to males (Luckinbill et al. 1988; Service 1989; Zwaan et al. 1995; Le Bourg and Minois 1996; Chippindale et al. 2004), suggesting that sexual dimorphism in longevity is context-dependent.

### Conclusions

There was little evidence of any effect of sexual conflict on investment in immune defence in our experiment, regardless of the type of conflict (IASC or IRSC). Nor did we find convincing evidence that longevity is subject to IASC in *Drosophila*, despite results from several other species where this is the case (reviewed in Adler and Bonduriansky 2014). However, we did find evidence that increased female control over mating rate had important and opposite effects on longevity between the sexes. Specifically, females which had adapted to high levels of female control over mating but were still occasionally exposed to wild-type males had high longevity when kept in mixed-sex groups. Conversely, males which had adapted to high levels of female control over mating had shorter longevity, perhaps due to increased investment in post-copulatory sexual selection (Manat et al. 2021). These results are novel because previous studies of the effect of IRSC on longevity have either focused on changes in longevity as a by-product of adaptation to novel environmental conditions (e.g. Arbuthnott et al. 2014; Duxbury et al. 2017), or else reduced the intensity of IRSC by manipulating adult sex ratios (e.g. Wigby and Chapman 2004; Nandy et al. 2013), which is likely to reduce both pre- and post-copulatory sexual selection. In contrast, although we did not manipulate mating rates directly, our experimental setup is likely to have reduced overall levels of sexual conflict in females and caused a shift towards the post-copulatory arena in males. Our results show that female control over mating rates may have important and unexpected effects on patterns of somatic investment, and therefore lifespan.

## Supplementary Information

Below is the link to the electronic supplementary material.

Supplementary file1 (DOCX 171 KB)

## Data Availability

All data and R scripts are available at https://github.com/KKLund-Hansen/FLXLongevityImmune.
