# Supplementary material for: Female-limited X chromosome evolution reveals that lifespan is mainly modulated by interlocus rather than intralocus sexual conflict
Source: Behav Ecol Sociobiol. 2022 Aug 17;76(9):120. doi: 10.1007/s00265-022-03231-4 (PMC9385781; doi:10.1007/s00265-022-03231-4)
Supplement: Supplementary file 1 — Supplementary file1 (DOCX 171 KB) [file 265_2022_3231_MOESM1_ESM.docx]

**Supplementary Information: Female-limited X chromosome evolution reveals that lifespan is mainly modulated by interlocus rather than intralocus sexual conflict**

Katrine K. Lund-Hansen^1^*, Megan A.M. Kutzer^2^, Sophie A.O. Armitage^3^, Samuel Gornard^1^, Hamilcar Keilani^1^, & Jessica K. Abbott^1^

*Corresponding author: [Katrine.Lund-Hansen@biol.lu.se](mailto:Katrine.Lund-Hansen@biol.lu.se)

1. Biology Department, Lund University, Sweden
2. Institute of Evolutionary Biology, University of Edinburgh, Scotland
3. Institute of Biology, Freie Universität Berlin, Germany

**Table 1** Summary of the ANOVA results for the linear mixed-models for resistance to infection and survival tolerance to L. lactis

| Source | | *df* | F | *P* |
| --- | --- | --- | --- | --- |
| Bacterial load | |  |  |  |
|  | Regime | 2 | 0.38 | 0.697 |
|  | Block | 1 | 0.22 | 0.641 |
|  | Nested factor | 1 |  | 0.506 |
| Survival tolerance | |  |  |  |
|  | Bacterial load | 1 | 1.45 | 0.236 |
|  | Regime | 2 | 0.05 | 0.947 |
|  | Block | 1 | 5.61 | *0.024* |
|  | Load × regime | 2 | 0.56 | 0.575 |
|  | Nested factor | 1 |  | 0.802 |

**Table 2** Summary of the ANOVA results for the Cox mixed-effects models for the flies when exposed to infection

| Source | | *df* | *χ^2^* | *P* |
| --- | --- | --- | --- | --- |
| Longevity 7 days | |  |  |  |
|  | Regime | 2 | 0.54 | 0.762 |
|  | Bacterium | 3 | 514.42 | *< 2e^-16^* |
|  | Block | 1 | 0.02 | 0.886 |
|  | Bacterium × Regime | 6 | 3.75 | 0.711 |
| Longevity 22 days | |  |  |  |
|  | Regime | 2 | 1.08 | 0.582 |
|  | Bacterium | 1 | 209.67 | *< 2e^-16^* |
|  | Block | 1 | 0.01 | 0.917 |
|  | Bacterium × Regime | 2 | 3.83 | 0.148 |
| Longevity Naïve 22 days | |  |  |  |
|  | Regime | 2 | 4.72 | 0.095 |
|  | Block | 1 | 0.07 | 0.798 |

**Table 3:** Cox Proportional Hazards for naïve flies for 22 days

|  | Hazard ratio | SE | z | P |
| --- | --- | --- | --- | --- |
| FLX |  |  |  |  |
| CFM | 0.49 | 0.87 | -0.82 | 0.410 |
| Cwt | 2.28 | 0.60 | 1.37 | 0.170 |

**Table 4** Summary of the non-significant ANOVA results for the Cox mixed-effects models for flies either isolated or in mixed-sex groups

| Source | | *df* | *χ^2^* | *P* |
| --- | --- | --- | --- | --- |
| Longevity female isolated | |  |  |  |
|  | Regime | 2 | 0.46 | 0.796 |
| Longevity male mixed-sex group | |  |  |  |
|  | Regime | 2 | 0.28 | 0.870 |

**Table 5:** Cox Proportional Hazards for females isolated

|  | | Median age (CI) | Hazard ratio | SE | z | P |
| --- | --- | --- | --- | --- | --- | --- |
| FLX | | 62 (61 – 65) |  |  |  |  |
|  | Replicate population 1 | 62 (59 – 69) |  |  |  |  |
|  | Replicate population 2 | 61.5 (58 – 68) |  |  |  |  |
|  | Replicate population 3 | 69 (62 – 73) |  |  |  |  |
|  | Replicate population 4 | 54 (50 – 60) |  |  |  |  |
| Control FM | | 62 (60 – 64) | 1.11 | 0.17 | 0.60 | 0.550 |
|  | Replicate population 1 | 64 (61 – 69) |  |  |  |  |
|  | Replicate population 2 | 58.5 (57 – 62) |  |  |  |  |
|  | Replicate population 3 | 65 (62 – 71) |  |  |  |  |
|  | Replicate population 4 | 60 (57 – 62) |  |  |  |  |
| Control wildtype | | 62 (59 – 66) | 1.11 | 0.18 | 0.56 | 0.570 |
|  | Replicate population 1 | 64 (53 – 71) |  |  |  |  |
|  | Replicate population 2 | 62 (55 – 69) |  |  |  |  |
|  | Replicate population 3 | 66 (62 – 71) |  |  |  |  |
|  | Replicate population 4 | 58 (55 – 69) |  |  |  |  |

**Table 6:** Cox Proportional Hazards for females in mixed-sex group

|  | | Median age (CI) | Hazard ratio | SE | z | P |
| --- | --- | --- | --- | --- | --- | --- |
| FLX | | 20 (18 – 23) |  |  |  |  |
|  | Replicate population 1 | 19.5 (13 – 26) |  |  |  |  |
|  | Replicate population 2 | 19 (15 – 24) |  |  |  |  |
|  | Replicate population 3 | 21 (17 – 33) |  |  |  |  |
|  | Replicate population 4 | 20 (17 – 29) |  |  |  |  |
| Control FM | | 27 (24 – 30) | 0.78 | 0.14 | -1.72 | 0.086 |
|  | Replicate population 1 | 18 (15 – 24) |  |  |  |  |
|  | Replicate population 2 | 30 (26 – 35) |  |  |  |  |
|  | Replicate population 3 | 31 (26 – 40) |  |  |  |  |
|  | Replicate population 4 | 33 (22 – 40) |  |  |  |  |
| Control wildtype | | 16 (13 – 20) | 1.17 | 0.15 | 1.01 | 0.310 |
|  | Replicate population 1 | 13 (8 – 18) |  |  |  |  |
|  | Replicate population 2 | 13 (12 – 23) |  |  |  |  |
|  | Replicate population 3 | 26 (20 – 33) |  |  |  |  |
|  | Replicate population 4 | 16 (13 – 31) |  |  |  |  |

**Table 7** Summary of the post hoc tests for the significant ANOVA results for the Cox mixed-effects models

|  |  | Estimate | Std. Error | z value | *P* |
| --- | --- | --- | --- | --- | --- |
| Longevity female mixed-sex group | |  |  |  |  |
|  | FLX – CFM | -0.24 | 0.142 | -1.72 | 0.199 |
|  | FLX – Cwt | 0.16 | 0.154 | 1.02 | 0.567 |
|  | CFM – Cwt | 0.40 | 0.154 | 2.59 | *0.026* |
| Longevity male isolated | |  |  |  |  |
|  | FLX – CFM | 0.29 | 0.122 | 2.35 | *0.049* |
|  | FLX – Cwt | -0.26 | 0.124 | -2.10 | 0.089 |
|  | CFM – Cwt | -0.55 | 0.125 | -4.39 | *< 0.001* |

**Table 8:** Cox Proportional Hazards for males isolated

|  | | Median age (CI) | Hazard ratio | SE | z | P |
| --- | --- | --- | --- | --- | --- | --- |
| FLX | | 55 (53 – 61) |  |  |  |  |
|  | Replicate population 1 | 61.5 (59 – 69) |  |  |  |  |
|  | Replicate population 2 | 60 (53 – 62) |  |  |  |  |
|  | Replicate population 3 | 62 (54 – 69) |  |  |  |  |
|  | Replicate population 4 | 46 (44 – 55) |  |  |  |  |
| Control FM | | 52 (50 – 54) | 1.36 | 0.19 | 1.59 | 0.110 |
|  | Replicate population 1 | 61 (54 – 69) |  |  |  |  |
|  | Replicate population 2 | 53 (50 – 62) |  |  |  |  |
|  | Replicate population 3 | 45 (41 – 55) |  |  |  |  |
|  | Replicate population 4 | 48 (43 – 53) |  |  |  |  |
| Control wildtype | | 62 (59 – 64) | 0.76 | 0.19 | -1.44 | 0.150 |
|  | Replicate population 1 | 62 (59 – 68) |  |  |  |  |
|  | Replicate population 2 | 55 (53 – 64) |  |  |  |  |
|  | Replicate population 3 | 62 (54 – 72) |  |  |  |  |
|  | Replicate population 4 | 65 (62 – 69) |  |  |  |  |

**Table 9:** Cox Proportional Hazards for males in mixed-sex group

|  | | Median age (CI) | Hazard ratio | SE | z | P |
| --- | --- | --- | --- | --- | --- | --- |
| FLX | | 37 (35 – 39) |  |  |  |  |
|  | Replicate population 1 | 35 (29 – 40) |  |  |  |  |
|  | Replicate population 2 | 40 (39 – 41) |  |  |  |  |
|  | Replicate population 3 | 37 (33 – 40) |  |  |  |  |
|  | Replicate population 4 | 33 (31 – 40) |  |  |  |  |
| Control FM | | 38 (36 – 40) | 0.92 | 0.19 | -0.44 | 0.660 |
|  | Replicate population 1 | 38 (35 – 43) |  |  |  |  |
|  | Replicate population 2 | 33 (30 – 38) |  |  |  |  |
|  | Replicate population 3 | 39 (35 – 40) |  |  |  |  |
|  | Replicate population 4 | 40 (32 – 41) |  |  |  |  |
| Control wildtype | | 37 (35 – 39) | 0.92 | 0.19 | -0.47 | 0.640 |
|  | Replicate population 1 | 37 (35 – 41) |  |  |  |  |
|  | Replicate population 2 | 41 (40 – 44) |  |  |  |  |
|  | Replicate population 3 | 33 (31 – 36) |  |  |  |  |
|  | Replicate population 4 | 38 (33 – 41) |  |  |  |  |

**Fig. 1** The experimental setup for the female-limited X chromosome evolution experiment. Female-limited X chromsome (FLX) selection regime: Every generation of X chromosomes are inherited from mother to daugther (red bar). By using an X chromosome balancer (FM, yellow bar), we are able to ensure this matrilineal inheritance while the X chromosome and FM balancer can not recombine. Control FM: We use the methological control regime to control for any unforseen effects of using the FM balancer. Unlike the FLX regime, every third generation the CFM X chromosome (green bar) are passed through males. For both regimes we added a recombination box, where the X chromosomes could recombine to amend genetic drift due to the smaller population size of the X. From Lund-Hansen et al. (2020).

**Fig. 2** Female immune defence. a Resistance to infection with L. lactis. We found no significant difference between the three regimes in resistance. Mean (±SE) of fitted values from the linear model. b Survival tolerance after infection with L. lactis. We found no significant difference in survival tolerance, which would have been indicated by a significant interaction between bacterial load and regime. FLX: light grey circle, CFM: dark grey square, Cwt: black triangle.

**Fig. 3** Survival after infection. a Survival after seven days for all four experimental treatments. We found no significant difference in survival between the three regimes in either of the four experimental treatments. b Survival after 22 days for naïve and P. entomophila injected flies. We found no significant difference between the three regimes in either of the two experimental treatments. c Survival only for naïve flies. When we only looked at naïve flies, we saw a tendency towards lifespan being different between the three regimes, which we followed-up in the longevity assay.

**Fig. 4** Lifespan for the four replicate populations in each of the three regimes in both sexes, either isolated or in mixed-sex groups. Black line: mean regime lifespan. Red line: replicate population 1, yellow line: replicate population 2, blue line: replicate population 3, and green line: replicate population 4. Each column is one sex either isolated or in mixed-sex groups, and each row is a selection regime. a FLX female lifespan when isolated. b FLX female lifespan in mixed-sex groups. c FLX male lifespan when isolated. d FLX male lifespan in mixed-sex groups. e CFM female lifespan when isolated. f CFM female lifespan in mixed-sex groups. g CFM male lifespan when isolated. h CFM male lifespan in mixed-sex groups. i Cwt female lifespan when isolated. j Cwt female lifespan in mixed-sex groups. k Cwt male lifespan when isolated. l Cwt male lifespan in mixed-sex groups.
